# Nanofiber/hydrogel core–shell scaffolds with three-dimensional multilayer patterned structure for accelerating diabetic wound healing

**DOI:** 10.1186/s12951-021-01208-5

**Published:** 2022-01-08

**Authors:** Jiankai Li, Tianshuai Zhang, Mingmang Pan, Feng Xue, Fang Lv, Qinfei Ke, He Xu

**Affiliations:** 1 Collaborative Innovation Center of Fragrance Flavour and Cosmetics, Shanghai Institute of Technology, No. 120 Caobao Road, Shanghai, 200235 People’s Republic of China; 2 College of Chemical and Materials Sciences, Shanghai Normal University, No. 100 Guilin Road, Shanghai, 200234 People’s Republic of China; 3 Department of Orthopedics, Shanghai Fengxian District Central Hospital, No. 6600 Nanfeng Road, Fengxian District, Shanghai, 201499 China

**Keywords:** Hydrogel/nanofiber scaffolds, Multilayer patterned structure, Angiogenesis, Cell infiltration, Diabetic wound healing

## Abstract

Impaired angiogenesis is one of the predominant reasons for non-healing diabetic wounds. Herein, a nanofiber/hydrogel core–shell scaffold with three-dimensional (3D) multilayer patterned structure (3D-PT-P/GM) was introduced for promoting diabetic wound healing with improved angiogenesis. The results showed that the 3D-PT-P/GM scaffolds possessed multilayered structure with interlayer spacing of about 15–80 μm, and the hexagonal micropatterned structures were uniformly distributed on the surface of each layer. The nanofibers in the scaffold exhibited distinct core–shell structures with Gelatin methacryloyl (GelMA) hydrogel as the shell and Poly (d, l-lactic acid) (PDLLA) as the core. The results showed that the porosity, water retention time and water vapor permeability of the 3D-PT-P/GM scaffolds increased to 1.6 times, 21 times, and 1.9 times than that of the two-dimensional (2D) PDLLA nanofibrous scaffolds, respectively. The in vitro studies showed that the 3D-PT-P/GM scaffolds could significantly promote cell adhesion, proliferation, infiltration and migration throughout the scaffolds, and the expression of cellular communication protein-related genes, as well as angiogenesis-related genes in the same group, was remarkably upregulated. The in vivo results further demonstrated that the 3D-PT-P/GM scaffolds could not only effectively absorb exudate and provide a moist environment for the wound sites, but also significantly promote the formation of a 3D network of capillaries. As a result, the healing of diabetic wounds was accelerated with enhanced angiogenesis, granulation tissue formation, and collagen deposition. These results indicate that nanofiber/hydrogel core–shell scaffolds with 3D multilayer patterned structures could provide a new strategy for facilitating chronic wound healing.

**Supplementary Information:**

The online version contains supplementary material available at 10.1186/s12951-021-01208-5.

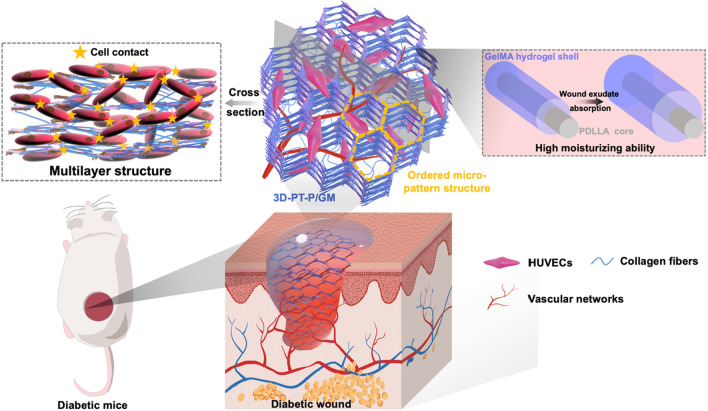

## Introduction

As a kind of serious complication of diabetes mellitus with high morbidity and risk of amputation, diabetic wounds have become a global health concern with the increase in the incidence of diabetes [1, 2]. Impaired angiogenesis in diabetic wound sites is one of the predominant reasons for those non-healing wounds [3]. Despite a lot of effort has been devoted to improving angiogenesis at the wound site, the results of existing therapies including growth factor treatment remain unsatisfactory in most cases [4, 5]. Therefore, pioneering an effective angiogenic therapeutic strategy for diabetic wounds is urgently needed.

Although skin tissue engineering proposes a promising way for diabetic foot ulcers, how to induce the rapid organization of endothelial cells (ECs) into a three-dimensional (3D) network of capillaries in the wound sites by using artificial scaffolds is still a long-standing challenge for tissue engineer [6]. Given the advantages of good biocompatibility and extracellular matrix (ECM)—like nanoscale structure, which could provide mechanical support and an ideal biomimetic environment for cell growth, electrospun nanofibrous scaffolds have been widely studied in wound healing [7–9]. Especially, compared with traditional electrospun scaffolds constructed with randomly deposited nanofibers [10], electrospun membranes with micro-patterned surface structure prepared by a simple collecting process show significant advantages in promoting vascularization [9, 11]. It has been proven that the ordered patterned structure in electrospun scaffolds such as fiber alignment can regulate endothelial cell alignment and body elongation, which can further induce cell migration and angiogenic differentiation [12, 13]. Therefore, the electrospun nanofibrous scaffold with micropatterned structure is a potential candidate which can be used to promote angiogenesis in diabetic wounds.

However, both the patterned and conventional non-woven electrospun scaffolds are facing the same challenge: the two-dimensional (2D) membrane-like structure with tightly packed nanofibers limit cell infiltration and growth throughout the scaffolds, and thus fail to induce ECs to form 3D vascular networks or direct cell migration from the bottom to the top for granulation tissue formation [14–16]. Studies have shown that scaffolds with orderly 3D network structure can act as more effective support for cell growth than 2D scaffolds by maintaining the uniform distribution of cells inside scaffolds and providing adequate levels of oxygen and nutrients, not only that, 3D porous scaffolds can recruit more giant cells and macrophages, which will be more conducive to assisting diabetic wounds to resist microbial damage [17, 18]. Therefore, it is of great importance to improve the porosity of 2D nanofibrous scaffolds in the third dimension while the micropatterned structures of those scaffolds could be maintained very well [16, 19–21].

To date, several methods have been reported to increase the porosity of nanofibrous scaffolds, including freeze–drying [22], template sacrifice [23], and gas-foaming [24]. However, most of these methods face the defects of uncontrollable pore size and thickness, complex preparation process, or toxic pore-forming agents. Among them, gas-foaming is a simple and facile approach for expanding traditional nanofiber membranes from 2 to 3D structures with controlled thickness and porosity [25]. Especially, the 3D nanofibrous scaffolds produced by the gas-foaming method exhibit regular layered structure which is more conducive to maintain the micropattern structure of scaffolds [26]. Compared with previous foaming methods, which use NH_4_HCO_3_ [27] and NaBH_4_ [28] for gas production, the subcritical CO_2_ fluid shows the advantages of a very short preparation period, room temperature, chemical inertness and non-toxic, etc. [17, 18]. Most importantly, this method can be used to fabricate water-soluble 3D scaffolds [18]. At room temperature, subcritical CO_2_ fluid will be changed into gaseous CO_2_ instantly by rapid depressurization. The scaffold dipped in subcritical CO_2_ fluid can be expanded into 3D nanofibrous scaffolds with multi-layer structure by the CO_2_ bubbles formed from instantaneous vaporization. Hence, the CO_2_ fluid was employed to prepare 3D nanofibrous scaffolds with multilayer patterned structures in this work.

It is critical that skin tissue engineering scaffolds could absorb a large amount of wound exudate and keep the moist environment of the wound [29]. Notably, a moist environment is favorable for cell survival and the release of growth factors at wound sites, which can further accelerate wound healing [30]. However, the conventional electrospun nanofibrous scaffolds always show poor hydrophilicity, for most of them are made of synthetic polymers, which leads to a dry microenvironment and failed cell adhesion [31]. Hydrogel is a kind of insoluble hydrophilic matrices with excellent biocompatibility, which can continuously absorb the exudate from the wound site and provide a moist microenvironment for the wound site [5]. Therefore, if we can design a nanofiber/hydrogel core–shell scaffold with nanofiber as the core and hydrogel uniformly coated on the surface of nanofiber as the shell, the hydrophilicity of the electrospun scaffold will be effectively improved, and this nanofiber/hydrogel core–shell scaffold will possess dual advantages of electrospun fiber and hydrogel such as ECM-like fibrous structure, high water retention, and excellent biocompatibility.

**Scheme 1:**
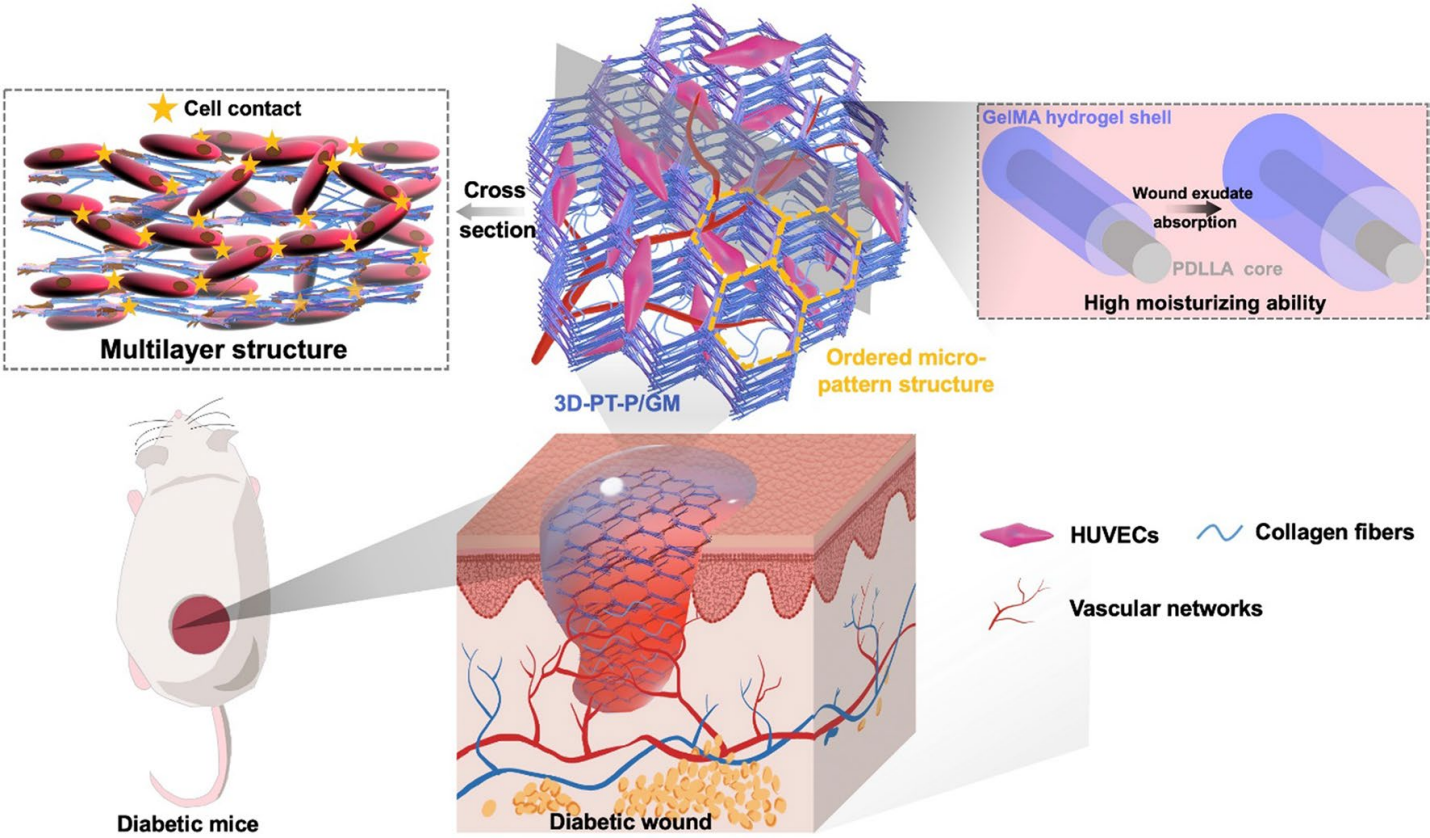
The core–shell hydrogel/nanofiber composite scaffold with 3D patterned structure for diabetic wound healing

Gelatin methacryloyl (GelMA) is a gelatin derivative modified by methacryloyl groups [32, 33]. It can form covalently crosslinked hydrogels through a UV photoinitiated radical polymerization with the presence of a photoinitiator while the unique properties of gelatins are retained [34]. A large number of arginine-glycine-aspartic acid (RGD) sequences in GelMA hydrogels make it excellent in promoting cell attachment and adhesion [32, 35]. Moreover, GelMA hydrogels have been reported to have the ability to promote the formation of vascular networks in vitro and in vivo [36]. Therefore, GelMA is an ideal candidate which can be coated on the surface of nanofibers and then form hydrogel in a photocrosslinking manner. Above all, we imagined to design a kind of core–shell hydrogel/nanofiber composite scaffolds with a 3D multilayer patterned structure, which possesses both outstanding water retention property and multilayer ordered porous structure. With the GelMA hydrogel coated on the surfaces of nanofibers, this scaffold could absorb the exudate in the wound site and provide a moist environment for wound repair. Especially, the 3D multilayer micropatterned structure and high porosity of the scaffold will promote cell infiltration, migration, and 3D vascular network formation, which eventually accelerate diabetic wound healing (as shown in Scheme 1).

**Fig. 1:**
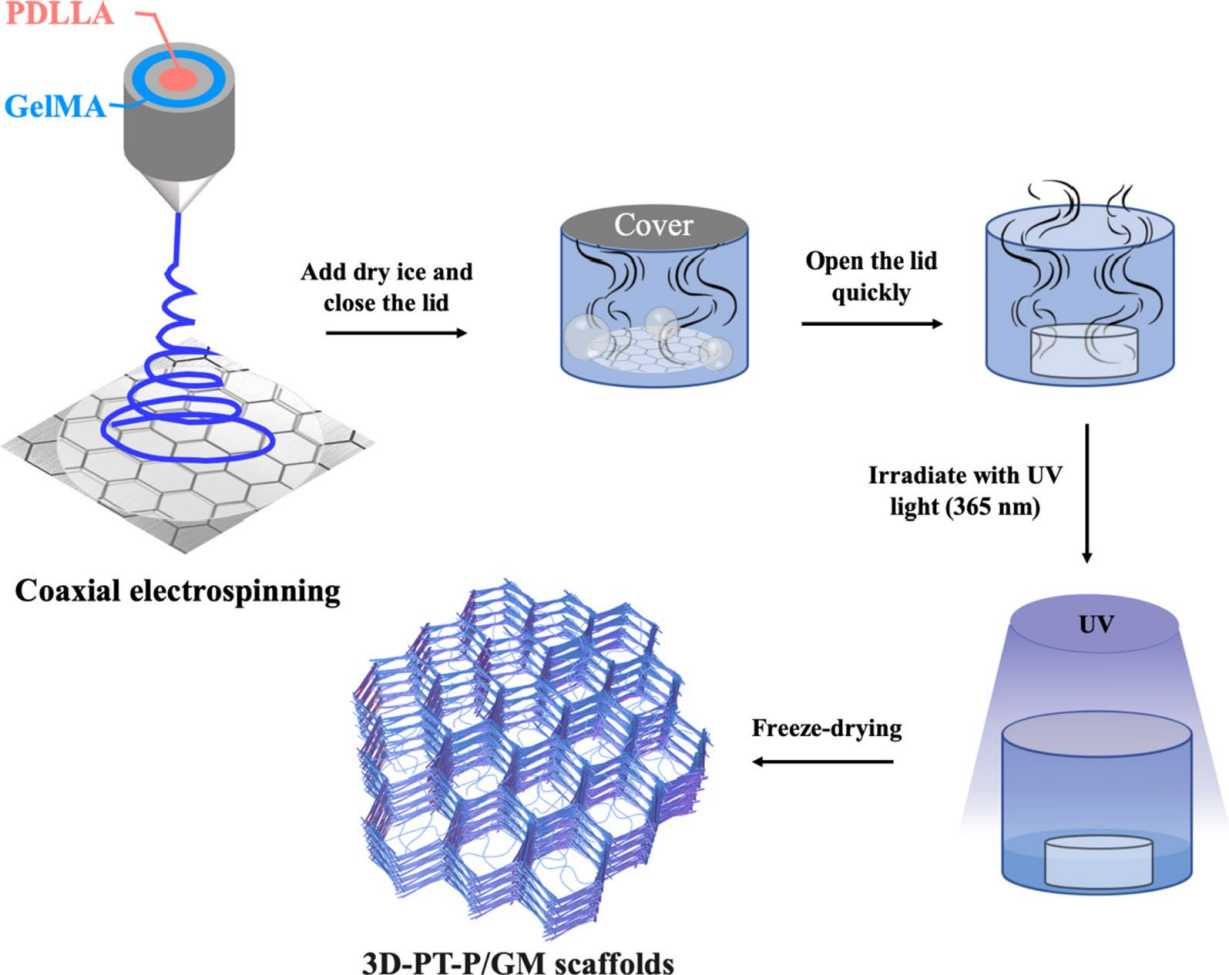
The preparation process of 3D-PT-P/GM scaffold

Herein, we firstly fabricated core–shell GelMA/Poly (d, l-lactic acid) (PDLLA) hydrogel/nanofiber scaffolds with 3D multilayer patterned structure (3D-PT-P/GM) for promoting diabetic wound healing. The GelMA/PDLLA hydrogel nanofiber was prepared by the coaxial electrospinning method (GelMA was the shell and PDLLA was the core), and the 3D multilayer patterned structure of the scaffold was obtained through the CO_2_ bubbles formed from instantaneous vaporization (as shown in Fig. 1). The morphology, porosity, water retention, and water vapor permeability properties of the fibrous membranes were systematically investigated. In vitro cell culture was carried out to verify that these scaffolds could promote cell infiltration, adhesion, proliferation, and migration. Besides, in vivo implantation was investigated to confirm the effects that these scaffolds could enhance angiogenesis, granulation tissue formation, and collagen deposition and further accelerate diabetic wound healing.

## Experimental

### Materials

Poly (d, l-lactic acid) (PDLLA, Mw = 800 kDa) was purchased from Jinan Daigang Biomaterial Co., Ltd. (Shandong, China). Gelatin (gel strength ~ 250 g Bloom), 1,1,1,3,3,3-hexafluoro-2-propanol (HFIP), methacrylic anhydride (MA), and 2-hydroxy-4′-(2-hydroxyethoxy)-2-methylpropiophenone (photoinitiator, Irgacure 2959) were supplied by Aladdin Reagent Co. (Shanghai, China). Dry ice was purchased from Shanghai Miteng Industrial Co., Ltd. (Shanghai, China). All materials were used without any further purification.

### Synthesis of gelatin methacryloyl (GelMA)

Gelatin methacryloyl (GelMA) was synthesized as reported in reference [32]. Typically, 1.0 g gelatin was dissolved in 10.0 mL phosphate buffered solution (PBS) at 50 °C for 1 h and stirred until forming a clear solution. 0.8 mL methacryloyl anhydride was slowly dropped into the above-stirred solution. After 3 h, the solution was added into 50 mL PBS (40 °C) under stirring for 15 min, and then the resulting solution was dialyzed against distilled water for a week at 40 °C (Mw cutoff 12,000–14,000 Da). Finally, the solution was lyophilized to get a white foam which was stored at − 20 °C until further use. ^1^H-NMR spectroscopy was used to detect the methacrylate groups grafted on the gelatin and the spectra were recorded in D_2_O on a Varian Mercury-400 MHz NMR spectrometer (Palo Alto, CA).

### Fabrication of nanofibrous scaffolds with micro-patterned structure

Nanofibrous scaffolds with micro-patterned structure were fabricated by electrospinning. In brief, 35.0 mg PDLLA was dissolved in 1.0 mL HFIP, and then the solution was fed through a syringe pump which was attached to a high-voltage statitron. The needle-to-collector distance was set to 15 cm. Nanofibrous scaffolds were electrospun at flow rates of 1.2 mL/h and voltages of 8 kV. To fabricate micro-patterned PDLLA nanofibrous scaffolds with a honeycomb structure, a hexagonal iron mesh was used as the receiving device of electrospun fibers. The collecting time of these micro-patterned PDLLA nanofibrous scaffolds was fixed at 2 h, and they were named as PT-P. The non-woven PDLLA nanofibrous scaffolds were collected as a control by using an iron plate collector for 2 h, which were named as RD-P. The PT-P and RD-P scaffolds were vacuum dried for 12 h at room temperature to remove residual HFIP.

### Fabrication of the core–shell hydrogel/nanofiber scaffolds with micro-patterned structure

Hydrogel/nanofiber scaffolds were fabricated by coaxial electrospinning technology. Briefly, 35.0 mg PDLLA and 70.0 mg prepared GelMA was dissolved in 1.0 mL HFIP respectively and then filled into two syringes as the core and shell precursor solutions. A hexagonal iron mesh (4 cm × 4 cm square plate) was used as receiving device and the needle-to-collector distance was set to 12–15 cm. During the electrospinning process, the core and shell precursor solutions were pumped at constant rates (1.0 mL/h and 1.2 mL/h, respectively) by glass syringes connected to the high voltage supplied with 10 kV. After being collected for 2 h, the scaffolds were vacuum dried for 12 h at room temperature to remove residual HFIP.

The GelMA in the shell of fibers was further cross-linked through a UV photoinitiated radical polymerization. Firstly, a photocrosslinking initiator solution was prepared by completely dissolving 1.0 g Irgacure 2959 in 10.0 mL ethanol solution in dark. Then, the prepared electrospun nanofibrous scaffolds were immersed in the photocrosslinking solution for 1 h, and then exposed to 365 nm UV light for 15 min. The crosslinked composite scaffolds were washed with ethanol and distilled water respectively to remove the residual photoinitiator and dried in a freeze dryer for 1 day. The obtained hydrogel/nanofiber scaffolds were named PT-P/GM.

### Fabrication of core–shell hydrogel/nanofiber scaffolds with 3D multilayer patterned structure

The uncrosslinked PT-P/GM nanofibrous scaffolds with micropattern were cut into 1 cm × 1 cm squares in liquid nitrogen. Subsequently, a piece of nanofibrous scaffold and 0.5 g of dry ice was placed in a well-sealed centrifuge tube. Once the dry ice was converted into a carbon dioxide fluid, the cap of the centrifuge tube was loosened quickly. After that, the centrifuge tube was placed in a dry environment. Finally, the uncrosslinked composite nanofibrous scaffolds with three-dimensional structures were cross-linked following the same protocol as that of PT-P/GM. The obtained cross-linked scaffolds were named as 3D-PT-P/GM.

### Characterization of core–shell hydrogel/nanofiber composite scaffolds with 3D multilayer patterned structure

#### Morphology and structure

The morphology of PT-P, PT-P/GM, and 3D-PT-P/GM scaffolds were determined by Digital camera (Samsung, Suwon Korea), field scanning electron microscopy (FE-SEM, Hitachi-S4800, CanScan) at accelerating voltage of 10 kV, and transmission electron microscopy (TEM, JEM-2100F JEOL, Japan) with accelerating voltage of 200 kV. The hydrophilicity of composite fibrous scaffolds was investigated by testing the water contact angle (WCA) on the surfaces (Kruss GmbH DSA 100 Mk 2). The chemical composition of the scaffolds was assessed by Fourier transform infrared spectroscopy (FTIR). Spectra were recorded on a Nicolet 6700 FT-IR spectrometer by the accumulation of 32 scans with a resolution of 4 cm^−1^ and a spectral range of 4000–500 cm^−1^. The chemical composition of scaffolds was also verified by ^1^H NMR (AVANCE 400 MHz).

#### Porosity

The porosity of RD-P, PT-P, PT-P/GM, and 3D-PT-P/GM scaffolds was calculated as reported in the previous studies [18, 37, 38]. The volume of nanofibrous scaffolds was recorded as V (V = L_(length)_ × W_(width)_ × T_(thickness)_). Length and width were tested by vernier caliper. Thickness was calculated according to the cross-section of the scaffolds shown in the SEM images. The volume of the bulk PDLLA material was calculated as V_P_ (V_p_ = m_p_/ρ_p_), m_p_ and ρ_p_ were the mass and density of nanofibrous scaffolds, respectively. The porosity (p) of RD-P and PT-P scaffolds were calculated according to the following Eq. (1):

1 $$p = \frac{{V - V_{p} }}{V} \times 100\% .$$

To evaluate the porosity of PT-P/GM and 3D-PT-P/GM scaffolds, the volume of the bulk GelMA material was calculated as V_GM_ (V_GM_ = m_GM_/ρ_GM_), m_GM_ and ρ_GM_ is the mass and density of the bulk GelMA material, respectively. Since the mass ratio of PDLLA to GelMA in the PGM fiber is 1:2. The volume of PGM (V_PGM_) was calculated as Eq. (2):

2 $$V_{PGM} = \frac{1}{3} \cdot m_{PGM} \cdot \frac{{2\rho_{p} + \rho_{GM} }}{{\rho_{p} .\rho_{GM} }}.$$

Finally, the porosity (p) of PT-P/GM and 3D-PT-P/GM were calculated according to the following Eq. (3):

3 $$p = \frac{{V - V_{PGM} }}{V} \times 100\% .$$

#### Water retention

Water retention of the RD-P, PT-P, PT-P/GM, and 3D-PT-P/GM scaffolds was calculated as described previously [39]. The weight of dry samples was recorded as W_Dry,_ and then the samples were immersed in distilled water. After 24 h, samples were left in the air and the weight (W_t_) was measured at pre-determined time points. The water sorption (SR) at t time was calculated according to the Eq. (4). Values are expressed as mean ± standard deviation (SD) (n = 3).

4 $$SR = \frac{{W_{t} - W_{Dry} }}{{W_{Dry} }} \times 100\% .$$

#### Water vapor permeability

Water vapor permeability of the RD-P, PT-P, PT-P/GM, and 3D-PT-P/GM scaffolds was calculated as described previously [39]. Samples were stuck around the mouth of 17 mm × 60 mm (O.D. × H) Glass Threaded Vials (Fisher, MA), which were filled with 6 mL PBS. The vials were placed in a shaker with a speed of 100 rpm (37 °C), and weighted every 24 h to determine the water loss. Values are expressed as mean ± standard deviation (SD) (n = 3).

### In vitro experimental

#### Cell proliferation assay

The human keratinocytes (HaCaTs), human umbilical vein ECs (HUVECs), and human dermal fibroblasts (HDFs) were purchased from the cell bank, Chinese Academy of Sciences. To evaluate the cell proliferation on the PT-P, PT-P/GM, and 3D-PT-P/GM scaffolds, the nanofibrous scaffolds were sheared into round pieces with a diameter of 10 mm and sterilized with 75% ethanol for 15 min for twice, then washed with sterilized PBS thrice. The HUVECs, HDFs, and HaCaTs were seeded on the surfaces of scaffolds (PT-P, PT-P/GM, and 3D-PT-P/GM) with a density of 6 × 10^3^ cells per well in 48-well culture plates (Corning, USA) respectively and maintained at 37 °C under a humidified atmosphere with 5% CO_2_. The viability of cells was measured by cell counting kit-8 (Kumamoto, Japan). The absorbance value of samples was measured at 450 nm using an enzyme-linked immunosorbent assay plate reader (Molecullar Devices).

#### Cell attachment and infiltration

To evaluate the cell attachment and infiltration on those scaffolds, the nanofibrous scaffolds were sterilized similarly to cell proliferation assay. HUVECs were seeded on the surfaces of PT-P, PT-P/GM, and 3D-PT-P/GM with a density of 2 × 10^4^ cells per well in 48-well culture plates (Corning, USA) and maintained at 37 °C under a humidified atmosphere with 5% CO_2_ for 24 h. Then, the cells were fixed with 4% (w/v) paraformaldehyde for 15 min. Finally, Cells were then permeabilized with 0.1% Triton-X100 for 10 min and incubated with FITC-phalloidin at 37 °C for 1 h. Cell attachment and infiltration could be observed by laser scanning confocal microscope.

#### Quantitative real-time polymerase chain reaction (QPCR)

To elaborate the mechanism related to the effects of PT-P, PT-P/GM, and 3D-PT-P/GM scaffolds on the differentiation of HUVECs, the angiogenesis-related gene expression of VE-Cad, N-Cad, VEGF, e-NOS, HIF-1α, and KDR was detected by quantitative real-time polymerase chain reaction (qPCR). HUVECs (4 × 10^4^ cells per well) were cultured on PT-P, PT-P/GM, and 3D-PT-P/GM scaffolds (n = 6) for 48 h, then, the total RNA was obtained and extracted by using the Trizol (Invitro- gen, Waltham, MA, USA). cDNA was synthesized from total RNA (1 μg) using Prime ScriptTM RT Master Mix (TakaraBio Inc., Shiga, Japan) at 37 °C for 30 min and 85 °C for 10 s. qPCR was conducted using SYBR Green detection reagent (Takara Bio Inc. Shiga, Japan). In this work, Actin was used as the reference gene and the primer sequences used for qPCR reactions were shown in Additional file 1: Table S1. All primers were found in the Primer Bank and synthesized by Genewiz (Shanghai), the specificity of the primers was confirmed before being used.

### In vivo experimental

#### Wound healing assessment

Female C57/BL6 mice with an average weight of about 18 g were obtained from the National Rodent Laboratory Animal Resources, Shanghai Branch of China, and all procedures were performed in compliance with the experimental protocols approved by the Animal Investigation Committee of the Institute of Biomedical Sciences and School of Life Sciences, East China Normal University.

The diabetic mice were induced by streptozotocin (STZ) according to previous reports [40]. Briefly, in order to induce diabetic mellitus-like symptoms, 6–8 weeks C57BL/6 mice were injected intraperitoneally with STZ (Sigma-Aldrich, St. Louis, MO) (50 mg/kg body weight per day) until the blood glucose levels were continuously higher than 20 mM. The blood glucose levels were measured by using glucose meters (Accu-Chek Performa). The induced diabetic mice were randomized into the Control, PT-P, PT-P/GM, and 3D-PT-P/GM groups according to their blood glucose levels and anesthetized with inhaled isoflurane (5%). After that, a standardized full-thickness wound with a diameter of 8 mm on the dorsum of each mouse was created. Then the three different scaffolds which were sterilized with 75% ethanol solution and washed thrice with sterilized PBS were applied to the wound beds. The wounds in the Control group were treated with no scaffold. At last, all the wounds were covered with breathable films (Tegaderm, 3M, USA). The images of the wounds were photographed on days 0, 3, 5, 7, 11 using a digital camera (Canon, Tokyo, Japan), and the remaining areas of the wounds were calculated by Image J.

Furthermore, to evaluate the new blood vessel formed in the wound site, the location of the wound tissues surrounding the wounds were collected after mice were sacrificed at 7 and 11 days post-surgery, respectively. The vascular infiltration state of each specimen was captured by a digital camera (Canon, Tokyo, Japan) and the newly formed blood vessels of the wound bed area were quantified.

#### Tissue specimen preparation and histological staining

The specimens of the skin tissue in the wound bed were collected at 7 and 11 days. Then those specimens were fixed with 4% paraformaldehyde for 24 h and dehydrated with a series of ethanol (from 50 to 100%), followed by being embedded in paraffin. The sections of the specimens were collected with a thickness of 5 μm. For observing the formation of collagen deposition and re-epithelialization at day 7, 11 post-surgeries, the sections were deparaffinized with dimethylbenzene, rehydrated with 100%, 95%, 80%, and 70% ethanol, and then stained using haematoxylin and eosin and Masson trichrome, respectively. The images were evaluated using an optical microscope (Leica Confocal microscope).

#### qPCR of the wound tissue

The total RNA of re-epithelized skin tissue was extracted by Trizol (Invitrogen, Waltham, MA, USA). Then, the isolated RNA (1 μg) was reversed transcribed into cDNA using Prime ScriptTM RT Master Mix (Takara Bio Inc., Shiga, Japan) at 37 °C for 30 min and 85 °C for 10 s. qPCR was conducted with SYBR Green detection reagent (Takara Bio Inc., Shiga, Japan). Actin was used as the reference gene and the primer sequences detected in this study were shown in Additional file 1: Table S2.

#### Immunohistochemistry staining

For the immunofluorescence staining, the wound tissue sections were rehydrated and boiled in sodium citrate buffer for 20 min and then incubated with primary antibody (CD31, Abcam, Cambridge, UK) overnight in an environment at 4 °C. Then the sections were incubated with secondary antibody for 2 h at room temperature. DAPI (40, 6-diamidino-2-phenylindole) was used for staining the nuclei. The images were evaluated using a confocal microscope (Leica Confocal microscope, Solms, Germany). The collagen area and CD31-positive vessel number were measured manually by Image J.

#### Assessment of tissue infiltration

Tissue infiltration level was observed by Masson trichrome staining. Briefly, areas of 4 cm^2^ on the back of three groups of mice were cut and each group of mice received implants of PT-P, PT-P/GM, and 3D-PT-P/GM scaffolds, respectively. Subcutaneous pockets were made through 1.5 cm incisions and each sample was compressed to 1.5 mm and inserted into the subcutaneous pocket by tweezer, and then the skin incisions were closed with staplers. After 30 days, mice were killed, and wound tissue specimens were obtained and stained by Masson trichrome using the same methods as 2.8.2.

### Statistical analysis

Three independent experiments were carried out and at least three samples per test were taken for statistical analysis. All data were presented as the arithmetic mean ± standard deviation. Statistical differences among more than two groups were calculated using one-way ANOVA firstly and then a student’s t-test program was further conducted to evaluate the significant difference between each two groups using GraphPad Prism 8. Differences were considered significant when p < 0.05 (*), p < 0.01 (**) or p < 0.001 (***).

## Results and discussion

### Fabrication of core–shell hydrogel/nanofiber composite scaffolds with 3D multilayer patterned structure

**Fig. 2:**
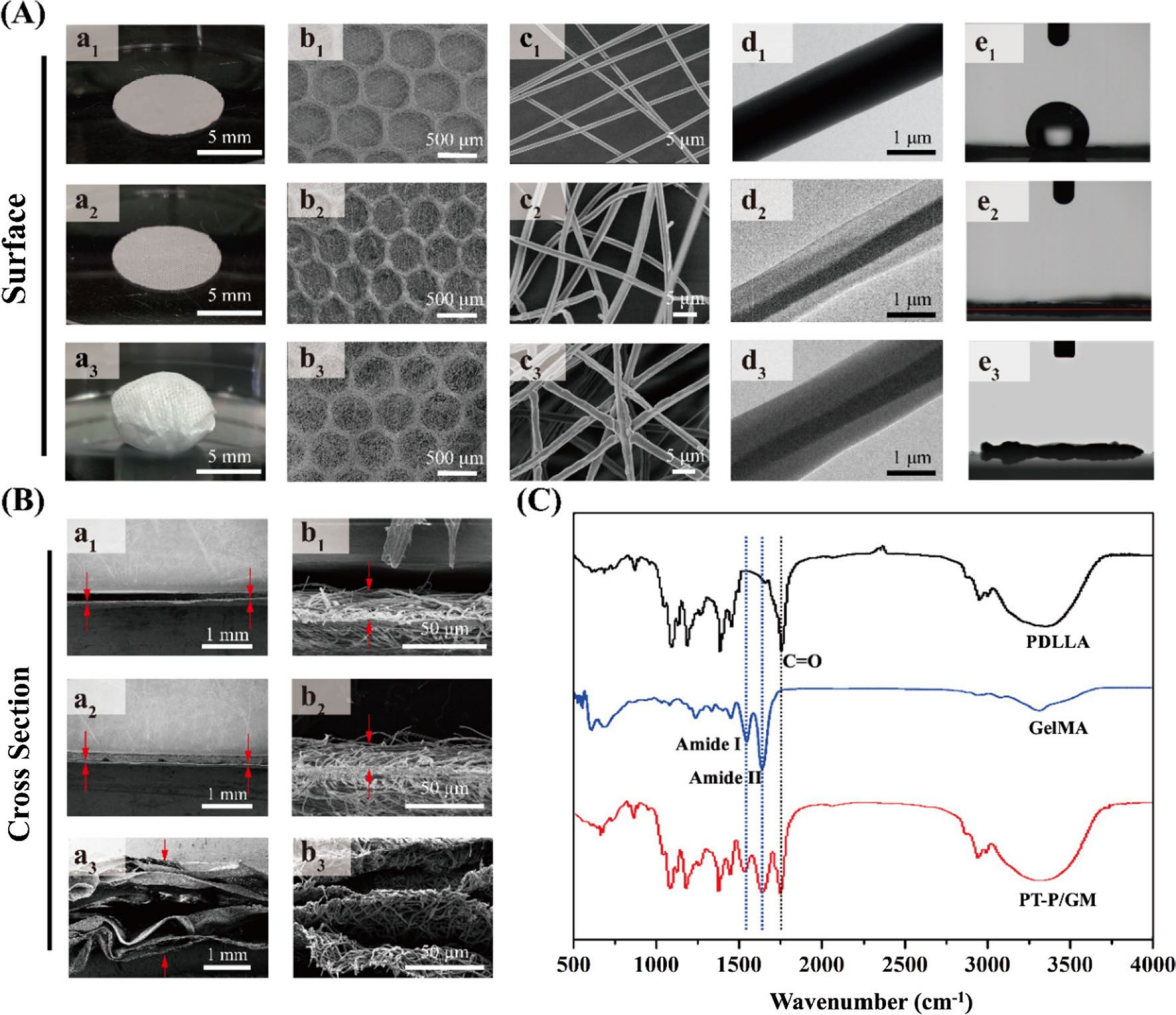
Characteration of the 3D hydrogel/nanofiber composite scaffolds. **A** Optical images (a_1_–a_3_), SEM images (low-magnification, b_1_-b_3_; High-magnification, c_1_–c_3_), TEM images (d_1_–d_3_) and water contact angle (e_1_–e_3_) for the three kinds of scaffolds: PT-P (a_1_–e_1_); PT-P/GM (a_2_–e_2_); 3D-PT-P/GM (a_3_–e_3_). **B** SEM images for the cross section of the three kinds of scaffolds: PT-P (a_1_–b_1_); PT-P/GM (a_2_–b_2_); 3D-PT-P/GM (a_3_–b_3_). **C** FTIR spectra of PDLLA, GelMA, PT-P/GM

As shown in the optical photographs (Fig. 2A (a_1_–a_3_)), both the PT-P (a_1_) and PT-P/GM (a_2_) samples showed typical 2D membrane structures, while the 3D-PT-P/GM (a_3_) showed a 3D fluffy structure with a thickness of about 8 mm. The corresponding SEM image in Fig. 2A (b_1_–b_3_, c_1_–c_3_) showed that all the three scaffolds exhibited similar micropatterned structures, the hexagonal patterns with a diameter of about 500 μm were distributed uniformly on the surfaces of the scaffolds (b_1_–b_3_). Most of the nanofibers deposited around the hexagon, while a few nanofibers deposited across the hexagon. Nanofibers in PT-P/GM (c_2_) and 3D-PT-P/GM (c_3_) scaffolds showed rougher surface than PT-P scaffold (c_1_), which might be due to the swelling effect of GelMA hydrogel and surface pultrusion effect of CO_2_ fluid.

From the TEM images (Fig. 2A (d_1_–d_3_)), it could be clearly observed that compared with the PT-P group, both the PT-P/GM (core diameter: 381 ± 8 nm, shell diameter: 386 ± 90 nm) and 3D-PT-P/GM (core diameter: 272 ± 28 nm, shell diameter: 340 ± 23 nm) possess distinct core–shell structures. The water contact angle (WCA) of the three kinds of scaffolds (Fig. 2A (e_1_–e_3_)) was investigated and the results revealed that compared with the PT-P scaffolds, both the PT-P/GM and 3D-PT-P/GM scaffolds exhibited excellent hydrophilic behaviors, which might be due to the hydrophilic GelMA hydrogel coated on the surfaces of nanofibers.

The cross-sections of the three scaffolds were shown in Fig. 2B. As observed, there was only a single layer constructed with tightly stacked fiber for both PT-P (a_1_, b_1_) and PT-P/GM (a_2_, b_2_) scaffolds, while the 3D-PT-P/GM (a_3_, b_3_) displayed a multilayered structure with the interlayer spacing of about 15–80 μm and the thickness of one single layer about 5 μm, which will offer enough space for cells growth inside scaffolds. All these results demonstrated that the nanofibrous scaffolds with 3D multilayer patterned structures had been successfully fabricated by the subcritical CO_2_ fluid method.

To confirm the structure of GelMA, ^1^H NMR spectrum analysis was conducted (Additional file 1: Fig. S1). The results showed that there were characteristic peaks of phenylalanine (at 7.1–7.4 ppm) appeared in both the spectrums of GelMA and gelatin. Previous studies have demonstrated that the unique biochemical properties of gelatin such as biocompatibility and abundant adhesion sites for cells were mainly attributed to the presence of phenylalanine [41, 42]. This result indicated that GelMA might possess similar characters to gelatin. Since the methacryloyl groups of MA will be grafted onto amino groups on the side chains of gelatin by the amidation reaction during the fabrication of GelMA [43], the spectrum results revealed that after grafting MA to gelatin, the characteristic peak of amino group at 2.9 ppm weakened, while the characteristic peaks of –C=CH_2_ on methacryloyl groups became obvious at 5.4 ppm and 5.6 ppm. Additionally, the characteristic peak of –CH_3_ in methacryloyl groups (at 1.7–1.9 ppm) could be also found in the spectrum of GelMA. These results indicated that the GelMA has been prepared successfully by the photocrosslinking method.

To further confirm the component of hydrogel/nanofiber scaffolds, the composition of GelMA/PDLLA hydrogel nanofibrous scaffolds was determined by FTIR spectrum (Fig. 2C). As observed, there was an obvious absorption peak appeared at 1758 cm^−1^ in the spectrum of PDLLA, which is corresponded to the C=O group [44], and the similar absorption peak was also appeared in the same position in the PT-P/GM spectrum. Not only that, two characteristic absorption peaks at 1627 cm^−1^ and 1528 cm^−1^, which are assigned to the characteristic peaks of amide I and amide II in gelatin, respectively, can be easily found in both the spectrums of GelMA and PT-P/GM. All these results indicated that the GelMA/PDLLA hydrogel nanofibrous scaffolds had been successfully fabricated though the combination of coaxial electrospinning and post-photocrosslinking technologies.

### Porosity, water retention, water vapor permeability characterization

**Fig. 3:**
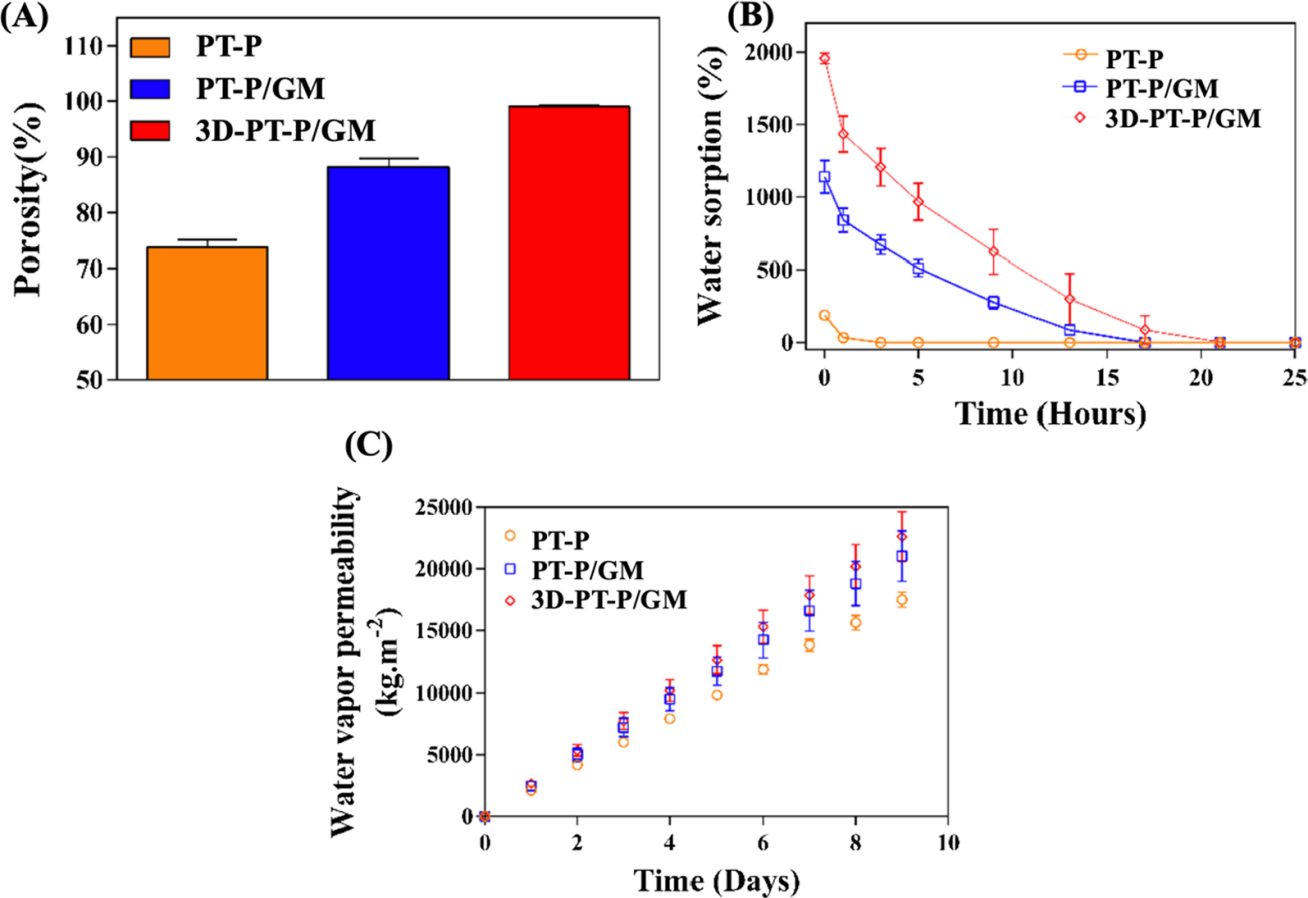
Characteration of the three kinds of scaffolds: PT-P, PT-P/GM, 3D-PT-P/GM. **A** Porosity. **B** Water retention ability. **C** Water vapor permeability

The porosity of the different kinds of scaffolds was studied according to the method reported in the previous references [18, 38]. As the results are shown in Additional file 1: Fig. S2A, the porosity of the PLLA nanofibrous membranes with fibers in random distribution (RD-P) was only 61.4%, while the porosity of PT-P increased to 73.7%, which may be due to the many hollow structures existed in the micro-patterned nanofibrous membranes. Notably, as shown in Fig. 3A, the porosity of PT-P/GM came up to 88.2%, which was attributed to the swelling effect of shell hydrogels leading to the increased curving degree of nanofibers. Compared with the traditional 2D electrospun nanofibrous membranes with nanofibers packed layer by layer tightly, which could only provide surface porosity, the 3D-PT-P/GM showed the largest porosity (99.1%), owing to the 3D multilayered structure that endowed the scaffolds pores in all directions.

As demonstrated in the previous studies, a scaffold that can provide an appropriate moist environment at wound sites is critical for the process of wound healing, for it can not only promote endothelial cell growth but also is favorable for cells to activate growth factors, which will further promote wound healing [45, 46]. The results of water retention ability of the scaffolds showed that, at the initial time, the 3D-PT-P/GM scaffolds absorbed almost 19.5 times of own weight water in PBS, while that for PT-P/GM, RD-P, and PT-P were 11, 2.46, and 2.55 times, respectively. Then, the water inside all the three scaffolds was evaporated gradually with the increase in time. The water inside 3D-PT-P/GM almost steamed out completely after 21 h, while the water inside PT-P/GM, RD-P, and PT-P was wholly evaporated after 17 h, 1 h, and 1 h, respectively. These results demonstrated that the 3D-PT-P/GM possessed both excellent water absorption capacity and water retention ability. Owing to the existence of GelMA, PT-P/GM and 3D-PT-P/GM had experienced a hydrophobic-to-hydrophilic transition and showed better water absorption than hydrophobic RD-P and PT-P. Notably, 3D-PT-P/GM possessed the best water retention capacity among all the scaffolds, it might be due to the large space between layers in the 3D-PT-P/GM scaffold which allows a large amount of water to be stored there.

Sufficient gas exchange and nutrition transportation are necessary for those cells growth inside scaffold during the wound healing process [47]. As an important indicator, the water vapor permeability of the scaffold was carried out in this work and the results showed that compared with the PT-P/GM (20.7 kg/m^2^) and PT-P (16 kg/m^2^) scaffolds, the 3D-PT-P/GM scaffold had the highest water vapor permeability with the value up to 22.3 kg/m^2^ at 9 days (Fig. 3C). The high porosity and 3D multilayered structure endowed the 3D-PT-P/GM with many pores and channels throughout scaffolds, which can benefit the transportation of gas and nutrition. In addition, it could be also observed that the water vapor permeability of PT-P scaffold (16 kg/m^2^) is much higher than that of RD-P scaffold (11.5 kg/m^2^), indicating that the pores created by the micropatterned structure in scaffolds could also facilitate the permeation of water vapor to a certain extent (Additional file 1: Fig. S2B).

### The effect of the 3D-PT-P/GM scaffolds on the cell adhesion, infiltration, proliferation and angiogenesis related gene expression in vitro

**Fig. 4:**
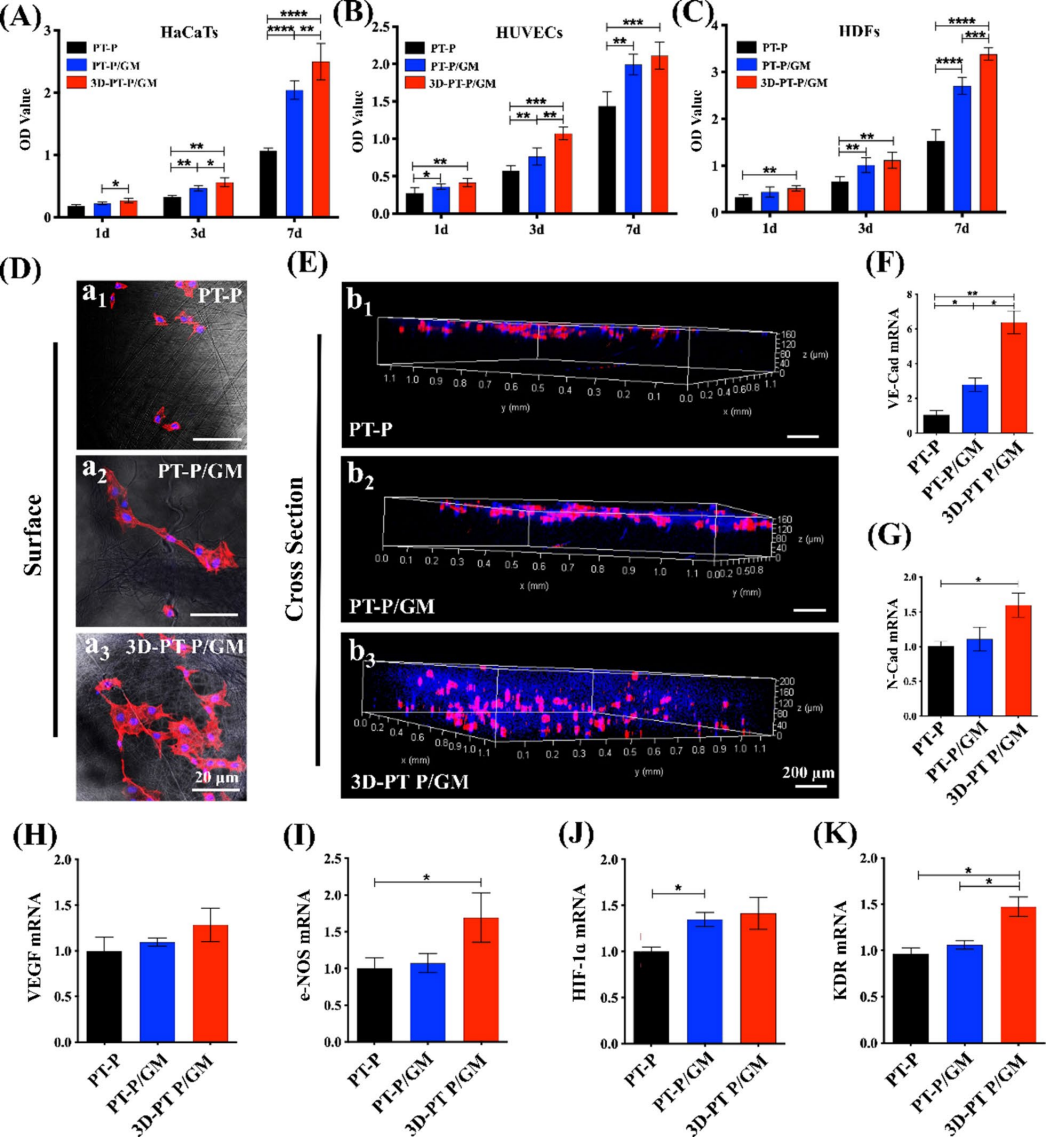
**A**–**E** Effect of the 3D hydrogel/nanofiber composite scaffolds on cell behavior in vitro. **A**–**C** The proliferation of the three kinds of cells in the different groups: **A** HaCaTs, **B** HUVECs, **C** HDFs. **D** HUVECs adhesion on the surfaces of the different kinds of scaffolds. **E** HUVECs infiltration through the scaffolds (blue, cell nucleus; red, cytoskeleton). **F**–**K** Quantitative analysis of VE-Cad (**F**), **G** N-Cad, **H** VEGF, **I** e-NOS, **J** HIF-1α, **K** KDR genes expressed in different groups. Values are expressed as mean ± standard deviation (SD) (n = 3) (*P < 0.05, **P < 0.01, ***P < 0.001)

To verify the effect of the 3D-PT-P/GM scaffolds on cell proliferation, HaCaTs, HUVECs, and HDFs were cultured on different scaffolds for 1, 3, and 7 days and the results were shown in Fig. 4A–C. HaCaTs, HUVECs, and HDFs showed excellent viability in all three kinds of groups, which indicated that all scaffolds had good biocompatibility and could support the growth of cells. Additionally, the number of the proliferative cells on PT-P/GM and 3D-PT-P/GM was significantly higher than that of the PT-P scaffolds at 1, 3, and 7 days, indicating that the presence of GelMA could promote cell proliferation. Previous studies have revealed that the surface wettability of scaffolds can affect the interaction between cells and scaffolds [48]. Compared to the hydrophobic PT-P scaffolds, cell adherence and vitality were greatly improved on the hydrophilic PT-P/GM and 3D-PT-P/GM scaffolds. Besides, it has also been demonstrated that the GelMA hydrogel on the surfaces of nanofibers can supplied active sites for cell adhesion. Hence, coating a GelMA layer on the surface of synthetic polymeric nanofiber can not only create a suitable hydrophilic environment but also provide abundant adhesion sites for cell growth. Notably, it could also be found that there were more proliferative cells in the 3D-PT-P/GM scaffolds when compared with that of PT-P/GM scaffold. It might be due to the 3D multilayered structure that was free from the space limitation that cells could only proliferate on the surface, the 3D multilayered structure provided abundant adhesion sites both surface and inside scaffolds, and cells could grow inside scaffolds through multilayered channels.

Since cell adhesion and infiltration throughout scaffolds plays a major role in the formation of 3D vascular networks, the cytoskeleton and nucleus of cells growth in the scaffolds were stained [49]. As shown in Fig. 4D (a_1_–a_3_), the HUVECs in 3D-PT-P/GM scaffold showed the best cell spreading and the most obvious aggregation and adhesion behavior among the three groups, while the HUVECs in PT-P scaffold showed the smallest area of cytoskeleton and nucleus, indicating that it is hard for HUVECs to adhere on PT-P scaffolds. The infiltration of HUVECs throughout the three scaffolds was analyzed by three-dimensional fluorescence imaging with a confocal microscope (Fig. 4E). As observed, most of the cells in PT-P/GM and PT-P groups were distributed only on the surface of the scaffolds, while more cells in 3D-PT-P/GM group were infiltrated throughout the scaffolds from the bottom to the top than the other two groups. Beside the sufficient RGD active sites and hydrophilic surface offered by GelMA hydrogel, which can facilitate cell adhesion and infiltration, the multilayered structure with the interlayer spacing of about 15–80 μm and the thickness of one single layer about 5 μm in the scaffold, can well support cell adhere and enter into the interlayer of the scaffold. Not only that, the patterned structure (with the diameter of about 500 nm) existed in each layer of the scaffold could allow cells infiltration across the layers. In this way, GelMA hydrogel and 3D multilayered structure of 3D-PT-P/GM promoted cell adhesion and infiltration throughout scaffolds synergistically.

It has been reported that favorable cell adhesion and infiltration conditions could enhance the communication among cells [50]. Vascular endothelial (VE)-cadherin and N-cadherin are two major junctional adhesion molecules that could mediate contact between adjacent ECs [51, 52]. In this work, QPCR was applied to quantitatively analyze the expression of cadherin genes (VE-Cad and N-Cad). As shown in Fig. 4F, G, the expression of the cadherin genes was significantly improved in the PT-P/GM and 3D-PT-P/GM groups, especially in the 3D-PT-P/GM group, in which both the two genes showed the highest expression levels. Previous studies have demonstrated that communication between adjacent ECs is one of the important events in angiogenesis [53]. ECs junctions are beneficial for maintaining endothelial integrity, regulating endothelial survival, and driving endothelial cell migration to form capillary [54]. In consistent with the previous studies, the results of angiogenesis-related genes (VEGF, e-NOS, HIF-α, and KDR) expression in Fig. 4H–K showed that the expression of all the four genes was significantly upregulated in 3D-PT-P/GM group. To sum up, these results confirmed the fact that the 3D-PT-P/GM scaffolds successfully promoted the proliferation, migration and especially infiltration of HUVECs and facilitated the angiogenic differentiation of HUVECs in vitro.

### The 3D-PT-P/GM scaffolds accelerating diabetic wound healing

**Fig. 5:**
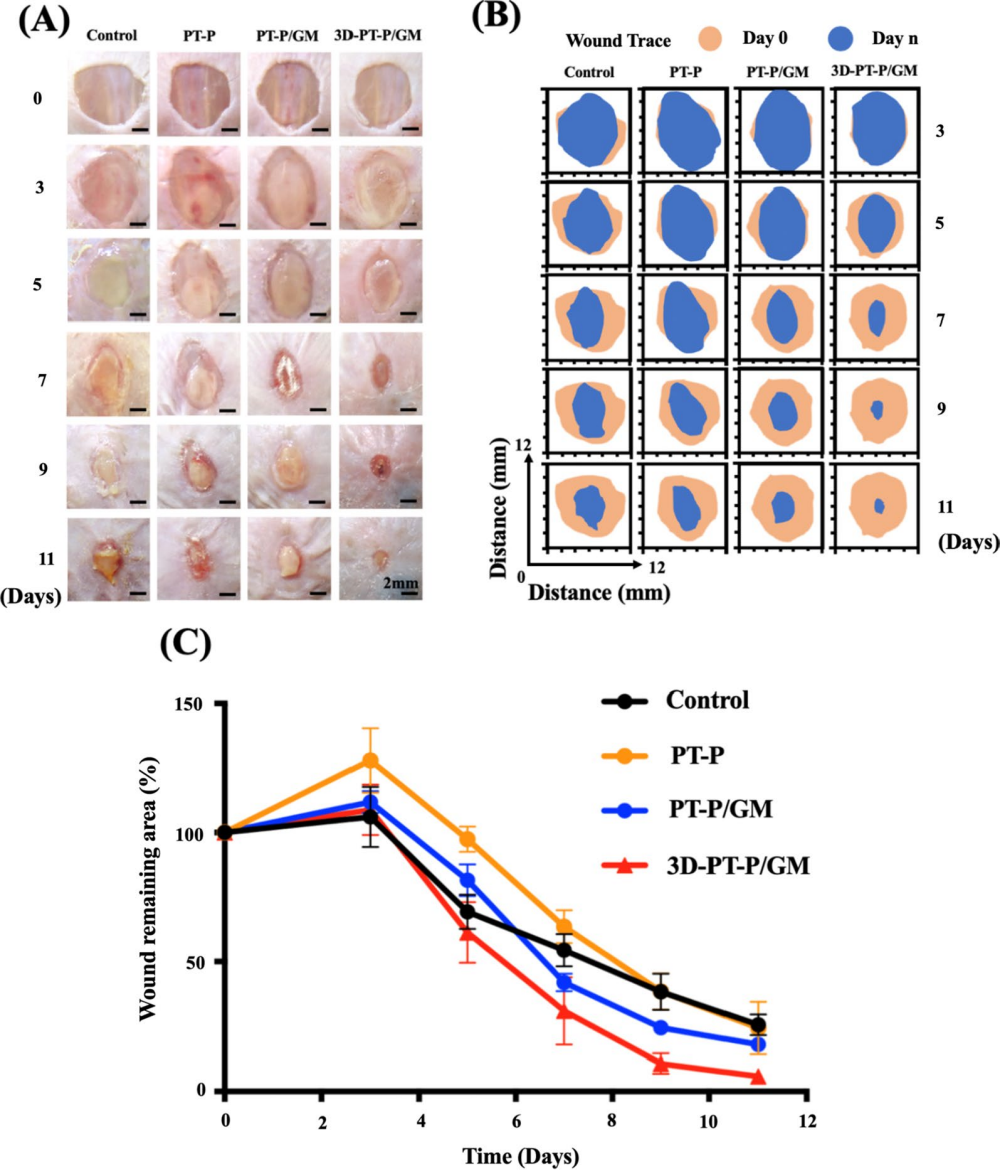
The effect of the 3D hydrogel/nanofiber composite scaffolds on diabetic wound healing. **A** Optical pictures of the wound areas for different groups on 0, 3, 5, 7, 9, 11 days post-surgery. **B** Schematic diagram of the remaining wound areas for different group in vivo (brown, wound areas on day 0; blue, wound areas on day n). **C** Statistical analysis of wound remaining areas in different groups on 0, 3, 5, 7, 9, 11 days post-surgery. Values are expressed as mean ± standard deviation (SD) (n = 3) (*P < 0.05, **P < 0.01, ***P < 0.001)

The effect of the 3D-PT-P/GM scaffolds on diabetic wound healing in vivo was investigated by using a STZ-induced diabetic mouse model. The optical images of the wounds of diabetic mice among four groups (Control, PT-P, PT-P/GM, and 3D-PT-P/GM) at 0, 3, 5, 7, 9, 11 days post-surgery were shown in Fig. 5A, respectively. Compared with the Control (54%) and PT-P (63%) groups, the PT-P/GM and 3D-PT-P/GM showed decreased average residual wound area of 42% and 34% at 7 days, respectively (Fig. 5B). In particular, the wound treated with 3D-PT-P/GM was approximately closed, while the Control, PT-P and PT-P/GM groups showed average residual wound area of 26%, 24%, and 18%, respectively (Fig. 5C). These results proved that 3D-PT-P/GM scaffolds could accelerate wound healing.

### The 3D-PT-P/GM scaffolds stimulating re-epithelialization

**Fig. 6:**
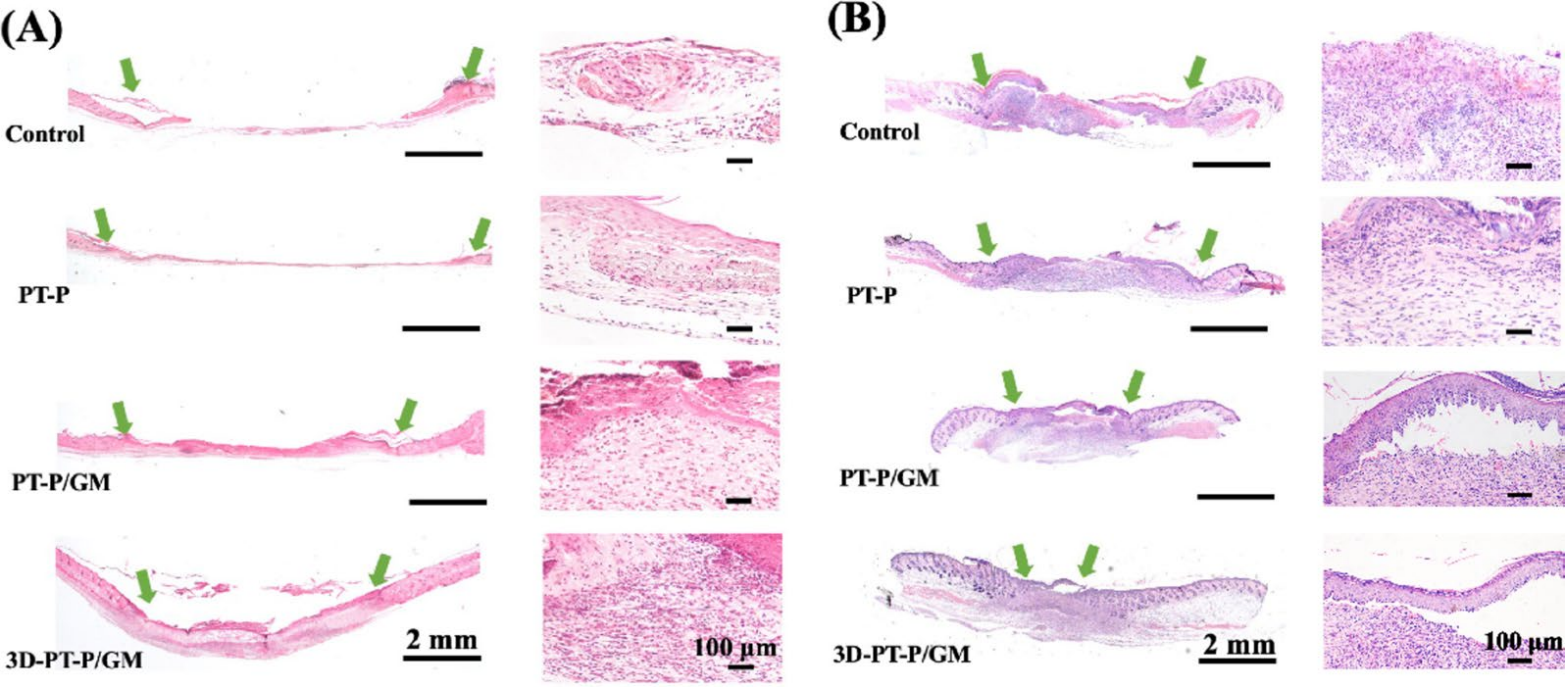
Effects of the 3D hydrogel/nanofiber composite scaffolds on re-epithelialization of wound beds. **A** H&E staining of wound beds on **A** 7 days and **B** 11 days (green arrows indicate the edges of the wounds; GT represented granulation tissue; NT represented neo-tissue; RE represented re-epithelialization tissue). Values are expressed as mean ± standard deviation (SD) (n = 3)

Re-epithelialization is a critical step for wound healing, which can form a barrier against foreign invasion [55, 56]. Epidermal cells proliferate and migrate to the wound surface and subsequently develop the complete epithelium [57]. In order to evaluate the re-epithelialization in the wound sites of the four groups, H&E staining assay of wound tissue at 7 days and 11 days was performed and the results were shown in Fig. 6A, B. As observed, the length of newly formed epithelium in PT-P/GM and 3D-PT-P/GM group treated group was significantly increased than that of the PT-P and Control at day 7. In addition, compared with the PT-P/GM group, the newly formed granulation tissue is more obvious in 3D-PT-P/GM group, in which group the almost healed epithelium could be observed after 11 days. Due to the abundant adhesion sites and hydrophilic surface offered by GelMA hydrogel, cell adhesion was enhanced on the PT-P/GM and 3D-PT-P/GM scaffolds. Especially, the transportation of oxygen and nutrition was significantly improved in the 3D-PT-P/GM scaffolds by the high porous 3D structure, cells could grow well inside the scaffolds, which could further accelerate the transformation of granulation tissue into neo-tissue along with the generation of epithelial tissue.

### The 3D-PT-P/GM scaffolds stimulating collagen deposition in diabetic wounds

**Fig. 7:**
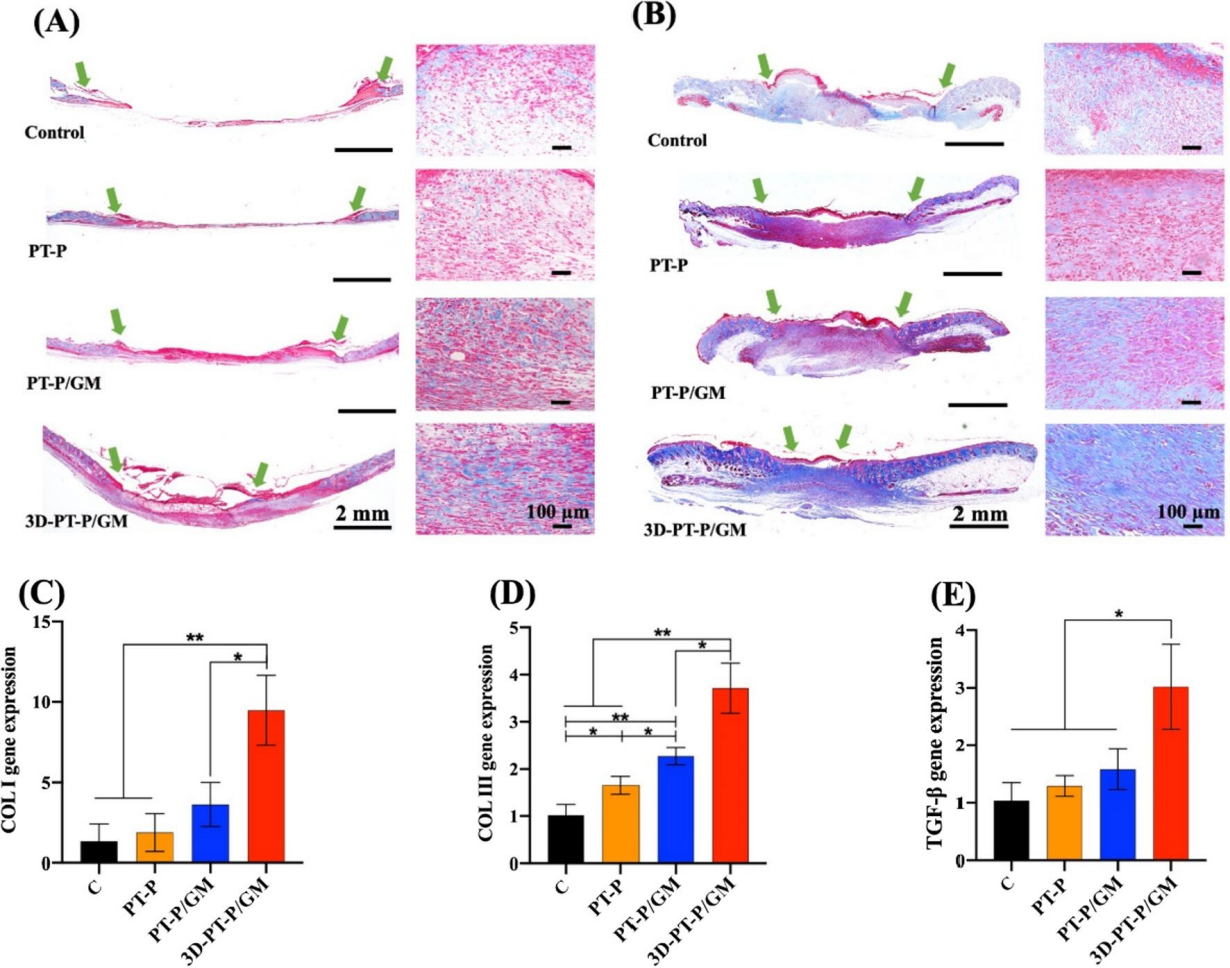
Effect of the 3D hydrogel/nanofiber composite scaffolds on collagen deposition in the wound beds. Masson trichrome staining images of wound tissues treated at **A** 7 days and **B** 11 days (green arrows indicate the edges of the wounds; blue areas indicate the collagen fibers). PCR gene quantitative analysis of **C** Collagen I, **D** Collagen III, **E** TGF-β expressed in different groups on 11 days. Values are expressed as mean ± standard deviation (SD) (n = 3) (*P < 0.05, **P < 0.01, ***P < 0.001)

Collagen fibers can not only enhance wound strength the during healing process, but also act as scaffolds to support cell adhesion, proliferation, and even differentiation [58]. To evaluate the deposition of collagen in diabetic wounds, Masson’s trichrome-staining assay was performed on the wound tissue at 7 and 11 days (Fig. 7A, B, respectively). The results showed that the collagen fibers (ECM remodeling marker, blue areas) appeared in all four groups increased with the increase of time, and the collagen fibers appeared in 3D-PT-P/GM group was significantly more than that in the other three groups at 7 days. Notably, 3D-PT-P/GM group possessed the deepest blue color and densest blue area among four groups at 11 days. Previous studies have demonstrated that a large amount of collagen was secreted during wound healing, among which collagen I and collagen III are the two main types of collagen, especially, the secretion of collagen I is conducive to remodeling the wound. Therefore, the production of collagen I and collagen III were assessed to detect the quality of the wound [59, 60]. In this work, the genes expression of collagen I and collagen III in 3D-PT-P/GM groups showed a much higher level than that of the Control, PT-P, and PT-P/GM groups (Fig. 7C, D, respectively), indicating the high quality of the healed wound. TGF-β can promote fibroblast accumulation into the wound site to produce collagen fibers and activate collagen I expression by phosphorylating the serine and threonine of regulatory proteins [61]. The results shown in Fig. 7E revealed that the 3D-PT-P/GM scaffolds significantly increased TGF-β genes expression at wound sites. To sum up, these results confirmed the fact that the 3D-PT-P/GM scaffolds could promote the deposition of collagen through the upregulation of TGF-β genes expression.

### The 3D-PT-P/GM scaffolds stimulating angiogenesis in diabetic wounds

**Fig. 8:**
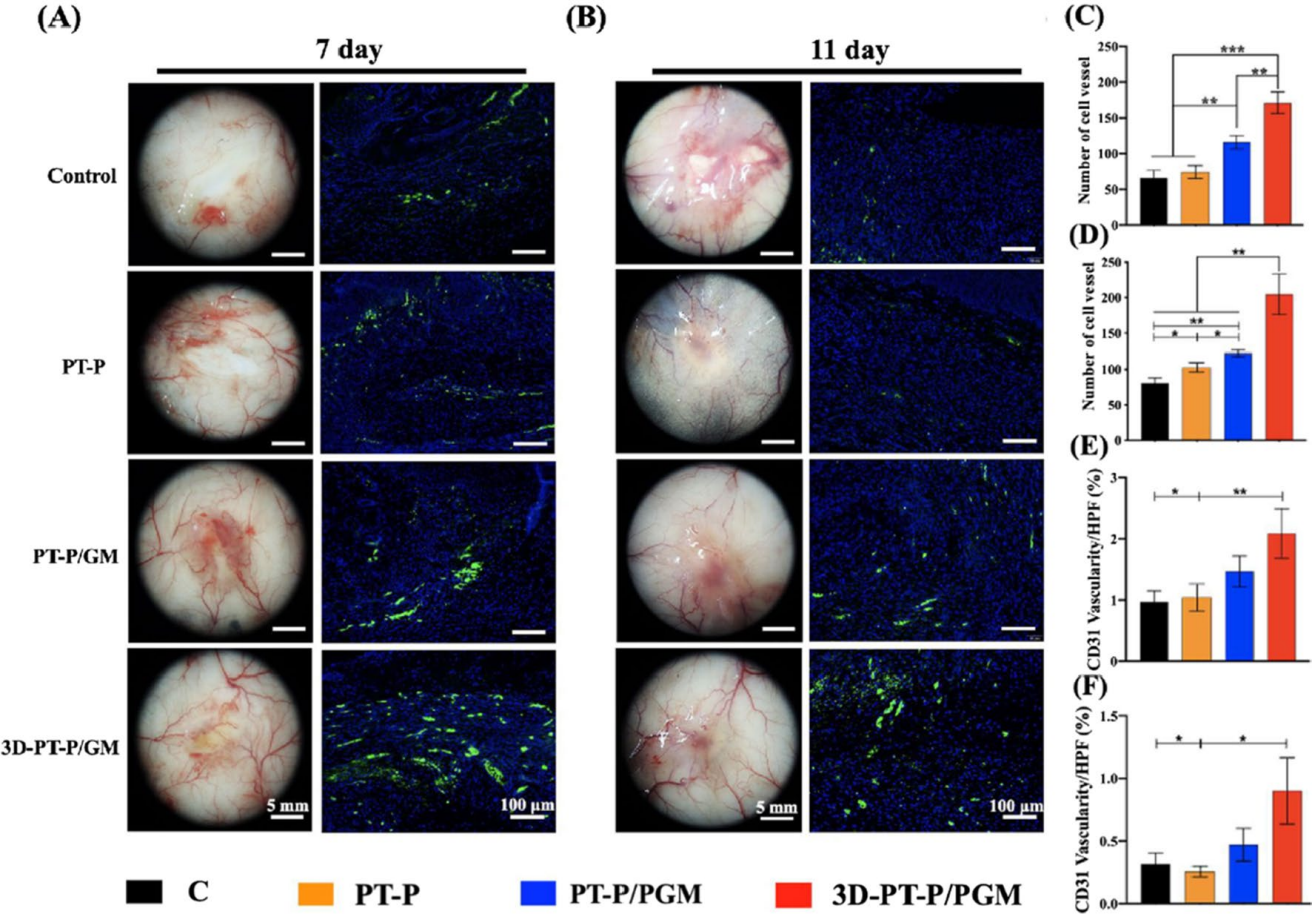
Effect of the 3D hydrogel/nanofiber composite scaffolds on the angiogenesis in the wound beds. **A** Representative images of vessel network formation and immunofluorescent staining analysis of CD 31 expression at 7 and 11 days, respectively. (Green areas, CD31 positive cells indicating blood vessels; blue, nuclei stained with DAPI). Quantitative analysis of vessel network formation at **C** 7 days and **D** 11 days. Quantitative analysis of CD31-positive vessels per high-power field at **E** 7 days and **F** 11 days. Values are expressed as mean ± standard deviation (SD) (n = 3). (*P < 0.05, **P < 0.01, ***P < 0.001)

In vitro experiments have demonstrated that 3D micro-patterned core–shell hydrogel nanofibrous scaffolds could enhance angiogenesis by stimulating the proliferation and intercellular communication of HUVECs. To investigate the effect of scaffolds on blood vessel formation in vivo, the stereomicroscope images of the newly-formed blood vessels in the diabetic wound sites of different groups at 7 and 11 days were taken, respectively. As shown in Fig. 8A, B, it could be seen obviously that more intensive blood vessels appeared in the PT-P/GM and 3D-PT-P/GM groups than that of the Control and PT-P groups. Especially, the 3D-PT-P/GM group exhibited an interconnected vascular network and the number of blood vessels in the 3D-PT-P/GM group was significantly the highest among all the groups at 7 and 11 days. To further confirm the newly-formed blood vessels in the wound sites, CD31 (a marker protein of angiogenesis) staining was carried out after 7 and 11 days post-surgery (Fig. 8A, B (right)). The results showed that CD31-positive (green fluorescence) area in the 3D-PT-P/GM group was the largest than that of the other three groups at 7 and 11 days. However, positive markers in all groups decreased at 11 days, which may be possibly due to the degeneration of capillaries after the wounds were healed. These results were further confirmed by the quantitative analysis of the number of blood vessels and CD31 staining at 7 and 11 days (Fig. 8C–F). Previous studies have demonstrated that the ordered micro-patterned structure could regulate cellular morphology, which is benefit for the induce of cell migration and angiogenic differentiation. The SEM images in Fig. 2 showed that the 3D-PT-P/GM scaffolds possessed multilayered structures, and there were micropatterned structures uniformly distributed on the surface of each layer. In this case, after infiltrating into the 3D scaffold, the cells will adhere on every fibrous membrane layer of the scaffold, both the GelMA hydrogel and micro-patterned structure could support cell well growth and regulate cellular morphology, which could influence cell angiogenic differentiation. More than these, the in vitro studies showed that besides facilitating the infiltration of ECs into the scaffolds, the 3D-PT-P/GM scaffolds promoted the communication between cells, which play an important role in the formation of the 3D vascular networks. Therefore, it could draw a conclusion that the 3D multilayered micropatterned structures of 3D-PT-P/GM scaffolds could significantly promote 3D vascular networks formation.

The cell infiltration in PT-P, PT-P/GM and 3D-PT-P/GM scaffolds in vivo were further evaluated by Masson staining. As displayed in Additional file 1: Fig. S4A, after 30 days, 3D-PT-P/GM scaffolds were filled with tissue cells and the middle part of the scaffolds had been degraded, while a large number of ordered collagen fibers as well as newly formed blood vessels were generated there. As shown in Additional file 1: Fig. S4B–D, the largest number of new-formed blood vessels was displayed in 3D-PT-P/GM scaffolds compared to other groups, and the results of CD31 staining also demonstrated that there were more CD31 positive areas appeared in 3D-PT-P/GM group than the other two groups. Combined with Masson trichrome staining in Additional file 1: Fig. S4A, it could be found that the new-formed blood vessels were mostly distributed in the infiltration area of tissue cells. All these results well confirmed the fact that 3D-PT-P/GM scaffolds significantly promoted the cell infiltration and accelerated the angiogenesis of HUVECs inside the scaffolds in vivo.

## Conclusion

In this work, a kind of nanofiber/hydrogel core–shell scaffolds with a three-dimensional multilayer patterned structure (3D-PT-P/GM) were successfully developed for promoting diabetic wound healing. The results indicated that the 3D-PT-P/GM scaffolds possessed high porosity up to 99.1%, high water retention (about 21 h), water absorption up to 1950%, and water vapor permeability up to 22.3 kg/m^2^. The in vitro experiments showed that GelMA hydrogel and 3D multilayer patterned structure can synergistically promote endothelial cell proliferation, migration, adhesion, infiltration, and angiogenic differentiation by upregulating the expression of cadherin (VE-Cad and N-Cad) and angiogenesis-related gene (VEGF, HIF-1α, e-NOS, and KDR). The in vivo studies further demonstrated that 3D-PT-P/GM scaffolds could significantly enhance cell infiltration, formation of vascular networks and collagen deposition, thereby ultimately accelerate the healing rate of diabetic wounds. This study suggests that the 3D-PT-P/GM scaffolds provide a new strategy for enhancing angiogenesis and promoting diabetic wound healing.

## Supplementary Information

**Additional file 1: Table S1.** The primer sequences used in the qPCR. **Table S2.** The primer sequences used for qPCR study in animal experiment. **Figure S1.** Schematic Illustration and ^1^H NMR spectra of the preparation of GelMA. **Figure S2.** Comparison between RD-P and PT-P for porosity and water vapor permeability. **Figure S3.** Quantitative analysis of Ki67 expressed in different groups on 11 days. **Figure S4.** Masson trichrome staining and immunofluorescence staining of wound beds for different post-surgery on 30 days. **Figure S5.** Agar disk diffusion assay in different groups.

## Data Availability

All data used to support the findings of this study are available from the corresponding author upon request.
