# Supplementary material for: Nanofiber/hydrogel core–shell scaffolds with three-dimensional multilayer patterned structure for accelerating diabetic wound healing
Source: J Nanobiotechnology. 2022 Jan 8;20:28. doi: 10.1186/s12951-021-01208-5 (PMC8742387; doi:10.1186/s12951-021-01208-5)
Supplement: Supplementary file 1 — Additional file 1: Table S1. The primer sequences used in the qPCR. Table S2. The primer sequences used for qPCR study in animal experiment. Figure S1. Schematic Illustration and 1H NMR spectra of the preparation of GelMA. Figure S2. Comparison between RD-P and PT-P for porosity and water vapor permeability. Figure S3. Quantitative analysis of Ki67 expressed in different groups on 11 days. Figure S4. Masson trichrome staining and immunofluorescence staining of wound beds for different post-surgery on 30 days. Figure S5. Agar disk diffusion assay in different groups. [file 12951_2021_1208_MOESM1_ESM.docx]

**Nanofiber/hydrogel core-shell scaffolds with three-dimensional multilayer patterned structure for accelerating diabetic wound healing**

*Jiankai Li ^b^, Tianshuai Zhang ^b^, Mingmang Pan ^c^, Feng Xue ^c^, Fang Lv ^c*^, Qinfei Ke ^a,b*^, He Xu ^b*^*

*^a^* Collaborative Innovation Center of Fragrance Flavour and Cosmetics, Shanghai Institute of Technology, No. 120 Caobao Road, Shanghai 200235, People’s Republic of China.

*^b^* College of Chemical and Materials Sciences, Shanghai Normal University, No.100 Guilin Road, Shanghai 200234, People’s Republic of China.

*^c^* Department of orthopedics, Shanghai Fengxian District Central Hospital No.6600 Nanfeng Road, Fengxian District, Shanghai, 201499, China.

**^*^ Corresponding authors:**

E-mail: xuhe@shnu.edu.cn (He Xu), kqf@shnu.edu.cn (Qinfei Ke), dafang_lucky@163.com (Fang Lv).

**Author Contributions：**

Jiankai Li and Tianshuai Zhang contributed equally.

**Additional file 1**

**Table S1.** The primer sequences used in the qPCR

| Gene | Forward Primer | Reverse Primer |
| --- | --- | --- |
| β- actin | 5’-GTACGCCAACACAGTGCTG-3’ | 5’-CGTCATACTCCTGCTTGCTG-3’ |
| VE-cad | 5’-AACCATGACAACACCGCC-3’ | 5’-CTCGTGAATCTCCAGCGC-3’ |
| N-cad | 5’-CGGTGCCATCATTGCCATCCT-3’ | 5’-AGTCATAGTCCTGGTCTTCTTCTTCT-3’ |
| VEGF | 5’-GAGCCTTGCCTTGCTGCTCTAC-3’ | 5’-CACCAGGGTCTCGATTGGATG-3’ |
| e-NOS | 5’-TGATGGCGAAGCGAGTGAAG-3’ | 5’-ACTCATCCATACACAGGACCC-3’ |
| HIF-1α | 5’-CACCACAGGACAGTACAGGAT-3’ | 5’-CGTGCTGAATAATACCACTCACA-3’ |

**Table S2.** The primer sequences used for qPCR study in animal experiment

| Gene | Forward Primer | Reverse Primer |
| --- | --- | --- |
| Collagen Ⅰ | CGCCATCAAGGTCTACTGC | ACGGGAATCCATCGGTCA |
| Collagen Ⅲ | CACCCTTCTTCATCCACT | ACAGATTATGTCATCGCAAA |
| Ki67 | ATCATTGACCGCTCCTTTAGGT | GCTCGCCTTGATGGTTCCT |
| TGF-β | AGCTGCGCTTGCAGAGATTA | AGCCCTGTATTCCGTCTCCT |


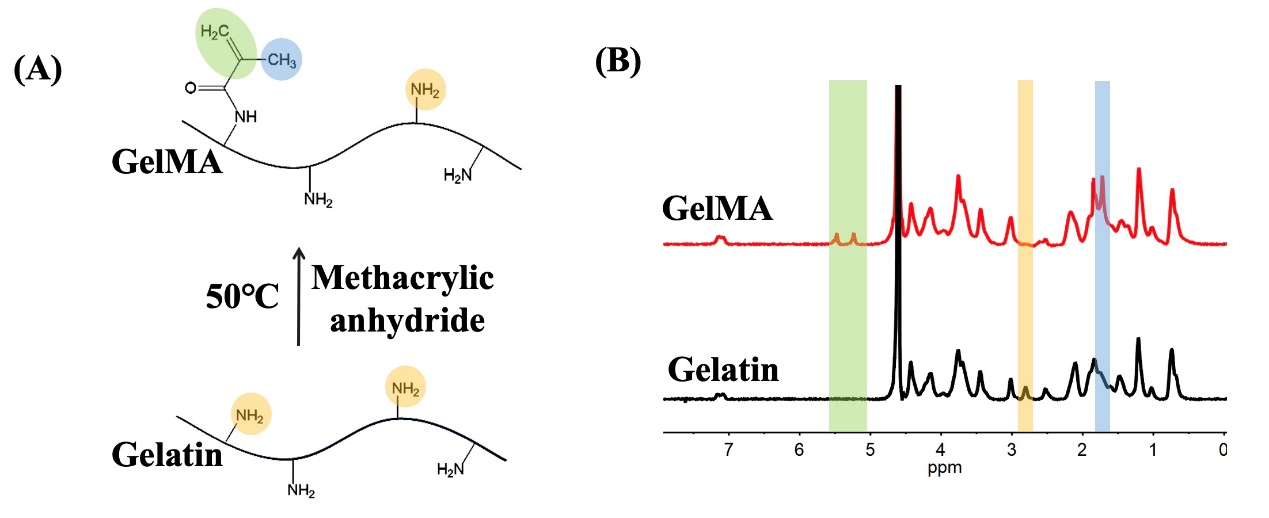


**Fig. S1 Preparation of GelMA. (A) Schematic Illustration of the Preparation of GelMA. (B) ^1^H NMR spectra of Geltain and GelMA.**


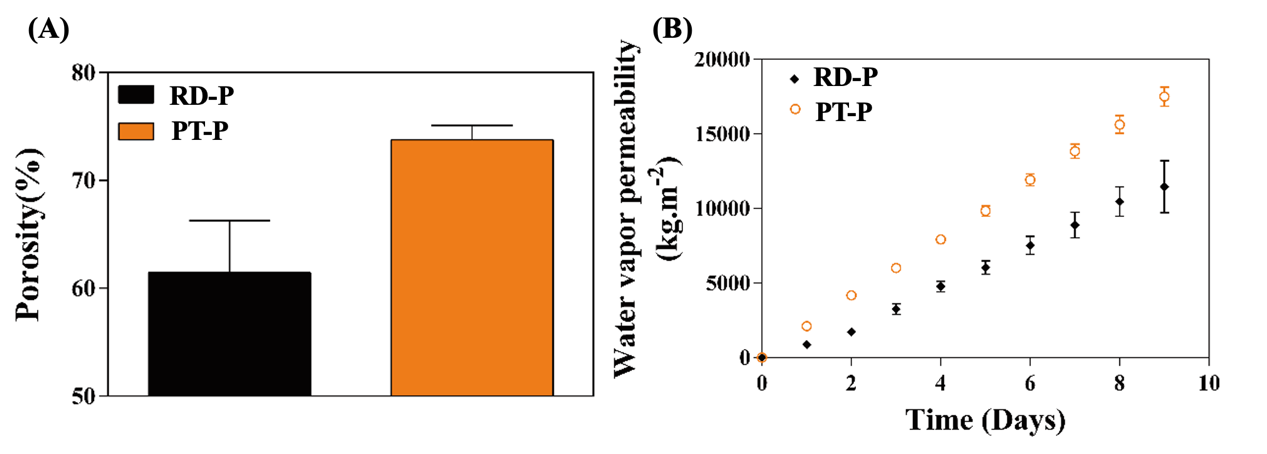


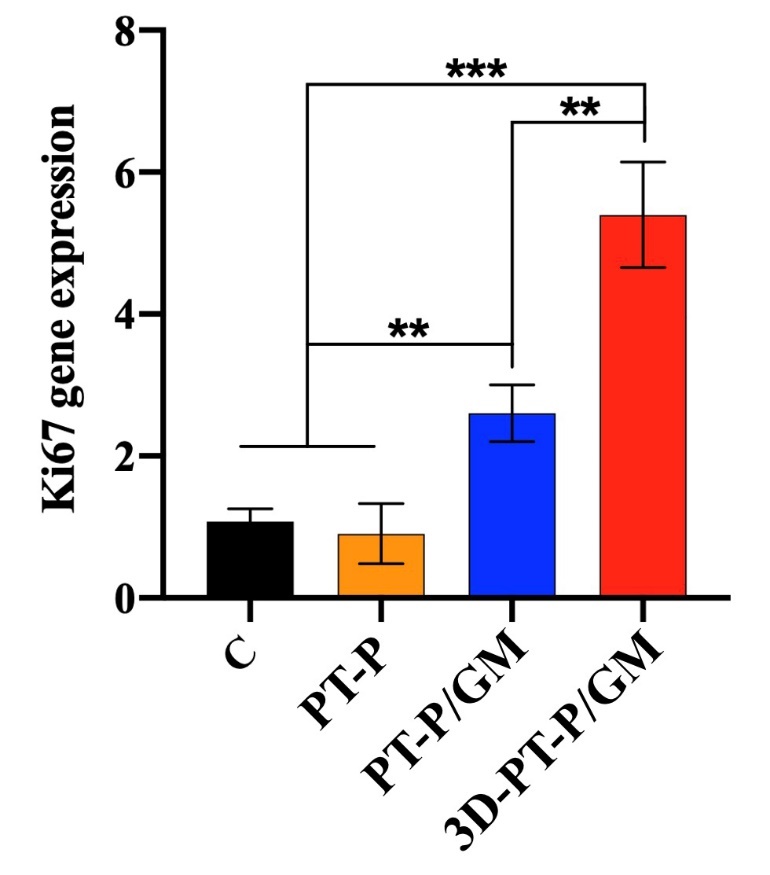
**Fig. S2 Comparison between RD-P and PT-P for porosity and water vapor permeability. (A) Porosity. (B) Water vapor permeability.**

**Fig. S3 Quantitative analysis of Ki67 expressed in different groups on 11 days. Values are expressed as mean ± standard deviation (SD) (n = 3) (*P < 0.05, **P< 0.01, ***P< 0.001).**


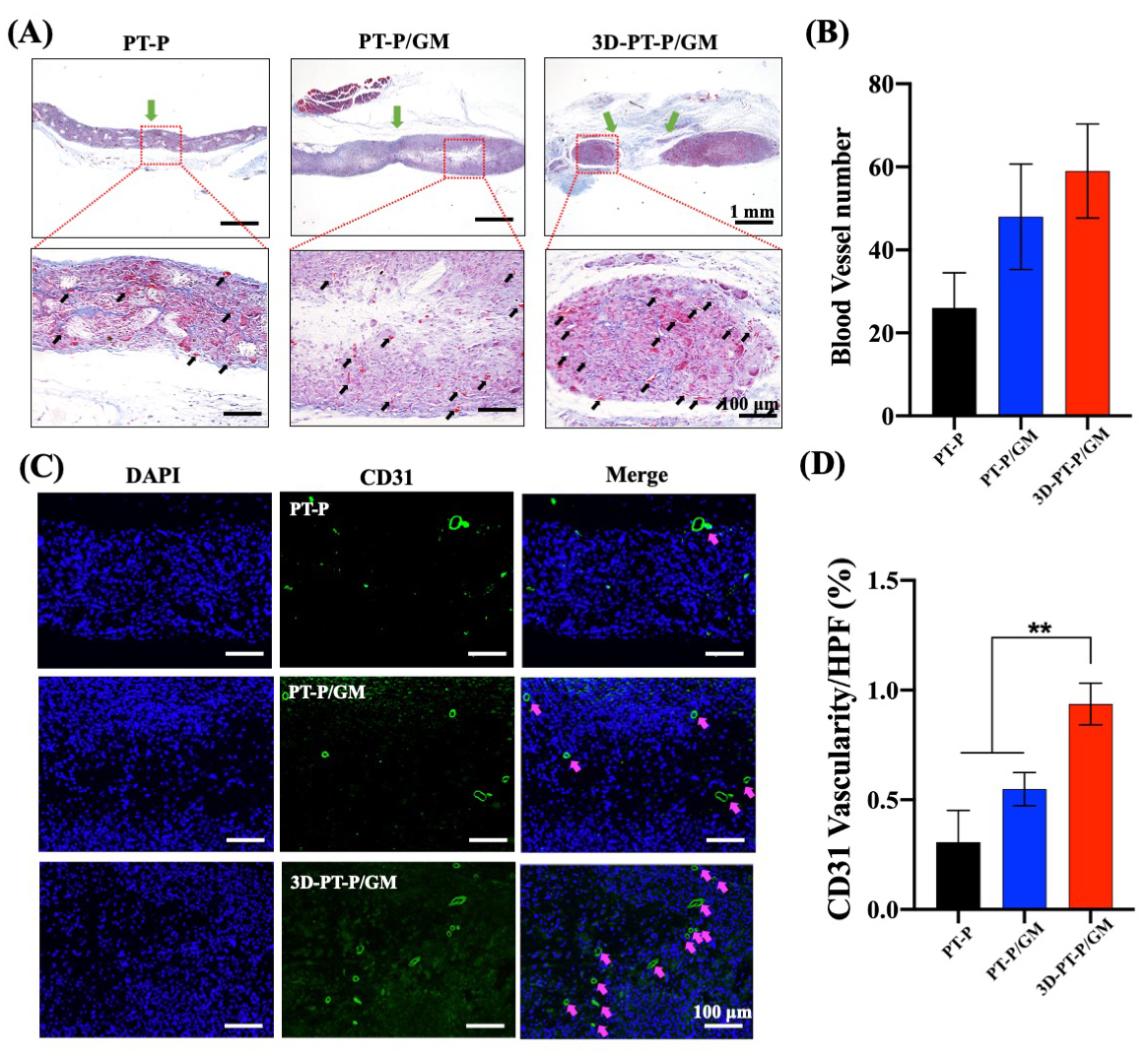


**Fig. S4 (A)** **Masson trichrome staining of wound beds for different post-surgery on 30 days (black arrows, blood vessels). (B) Quantitative analysis of vessel formation of wound beds on 30 days. (C)** **Immunofluorescence staining images of CD31 of wound beds on 30 days (pink arrows, CD31-positive vessels). (D) Quantitative analysis of CD31-positive vessels per high-power field on 30 days. Values are expressed as mean ± standard deviation (SD) (n = 3) (*P < 0.05, **P< 0.01, ***P< 0.001).**


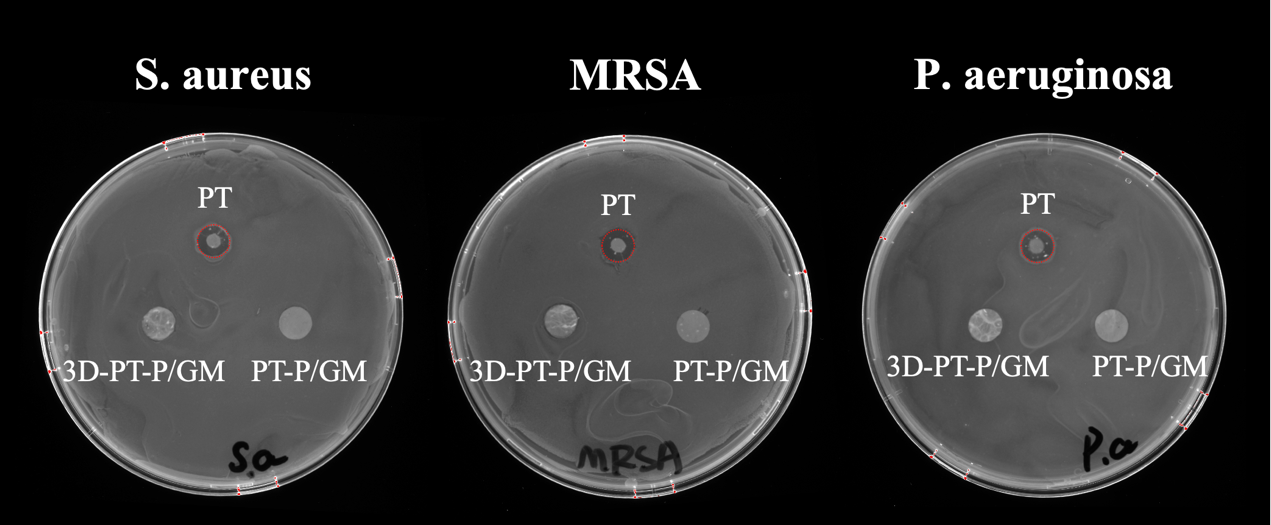


**Figure S5 Agar disk diffusion assay of PT, PT-P/GM and 3D-PT-P/GM scaffolds. (Three scaffolds with a diameter of 8 mm were placed in the culture medium of three different bacteria and incubated for 12 hours. Due to the high temperature, the PT scaffolds contracted).**
